# Suitability of Japanese Medical Databases for Studies on Infant Outcomes After Maternal Drug Exposure: An Evaluation Based on Core Data Elements

**DOI:** 10.1002/pds.70264

**Published:** 2025-11-12

**Authors:** Shiro Hatakeyama, Takamasa Sakai, Masami Tsuchiya, Daisuke Kikuchi, Yuri Sato, Yuki Kondo, Izumi Sato, Yuko Okada, Taku Obara

**Affiliations:** ^1^ Department of Pharmacy Tohoku Medical and Pharmaceutical University Hospital Sendai Miyagi Japan; ^2^ Department of Pharmacy Yamagata University Hospital Yamagata Japan; ^3^ Drug Informatics, Faculty of Pharmacy Meijo University Nagoya Aichi Japan; ^4^ Division of Drug Informatics Keio University Faculty of Pharmacy Tokyo Japan; ^5^ Division of Community Medicine and Pharmacy, Faculty of Pharmaceutical Sciences Tohoku Medical and Pharmaceutical University Sendai Miyagi Japan; ^6^ KTS Plan Inc. Sendai Miyagi Japan; ^7^ Department of Clinical Chemistry and Informatics, Graduate School of Pharmaceutical Sciences Kumamoto University Kumamoto Japan; ^8^ Department of Clinical Epidemiology, Graduate School of Biomedical Sciences Nagasaki University Nagasaki Japan; ^9^ Faculty of Pharmacy Takasaki University of Health and Welfare Takasaki Gunma Japan; ^10^ Department of Pharmaceutical Sciences Tohoku University Hospital Sendai Miyagi Japan

**Keywords:** database, perinatal medication, pharmacoepidemiology, pregnancy

## Abstract

**Purpose:**

Pharmacotherapy during pregnancy should be approached with caution due to the potential risk of adverse effects, including birth defects, in the fetus. Appropriate post‐marketing surveillance and perinatal pharmacoepidemiology are essential to ensure the safety of pharmacotherapy during pregnancy. However, due to limited research infrastructure in pharmacoepidemiology, collecting reliable data on drug safety in pregnant women and infants remains a challenge in Japan. Thus, we examined the suitability (fitness for purpose) of Japanese medical databases for perinatal studies to establish infrastructure in this field.

**Methods:**

We assessed seven available databases in Japan: the DeSC database, EBM provider, Japanese Adverse Drug Event Report database, JMDC claims database, MDV analyzer, the National Database of Health Insurance Claims, and Tohoku Medical Megabank Project Birth and Three‐Generation Cohort Study (TMM BirThree Cohort Study). To assess the quality of databases for maternal studies, we evaluated the content of each mother‐infant linkable database based on the core data elements recommended for perinatal pharmacoepidemiology.

**Results:**

Three of the databases were found to be mother‐infant linkable: the DeSC database, JMDC claims database, and the TMM BirThree Cohort Study. The coverage of core data elements in perinatal pharmacoepidemiology was 73.5% in the DeSC and JMDC claims databases and 92.9% in the TMM BirThree Cohort Study.

**Conclusion:**

Some representative medical databases in Japan are well suited for use in perinatal pharmacoepidemiologic research on infant outcomes.

**Summary**

- There is limited information available in Japanese medical databases for analyzing the association between maternal drug exposure and adverse effects on the fetus.
- We assessed the suitability of seven representative Japanese medical databases to perinatal studies by referencing core data element (CDE) variables for standardized data collection.
- Two administrative healthcare databases and one birth cohort database are useful for linking maternal and infant data, as they include a high proportion of relevant CDE variables.

## 1 Introduction

Appropriate post‐marketing surveillance and perinatal pharmacoepidemiology have been encouraged to ensure the safety of pharmacotherapy during pregnancy [1, 2, 3, 4]. However, significant concerns regarding perinatal drug safety, including the association between maternal drug exposure and congenital anomalies, are increasingly being raised in clinical practice and observational studies, as randomized clinical trials are generally not feasible in pregnant women for ethical reasons. Furthermore, the reliability of the available data sources remains uncertain [5].

To ensure the reliability of medical databases for use in perinatal pharmacoepidemiology, it is essential that they include a mother–infant linkage identifier and accurate information on the pregnancy period. A suitable database for evaluating drug safety during pregnancy [6] must also contain high‐quality and reliable data.

To address these concerns, core data element (CDE) variables were developed for standardizing data collection as part of the Innovative Medicines Initiative (IMI) ConcePTION project. These CDE variables are available on the European Network of Teratology Information Services (ENTIS) website and are used to assess the suitability of the database for perinatal pharmacoepidemiology [7]. Several medical databases used in perinatal pharmacoepidemiology have been reported to align closely with these CDE variables [7].

In contrast, relatively few medical databases in Japan are suitable for perinatal pharmacoepidemiologic studies focusing on infant outcomes following maternal drug exposure [8, 9, 10, 11]. The potential of Japanese medical databases as appropriate data sources for evaluating perinatal drug safety remains uncertain, as no published studies have yet confirmed the presence of mother–infant linkage identifiers or detailed pregnancy duration information. Therefore, we assessed the suitability (fitness for purpose) of existing Japanese medical databases as tools for perinatal pharmacoepidemiology of infant outcomes using the presence of CDE variables as the primary criterion.

## 2 Methods

### 2.1 Study Design

The suitability of Japanese medical databases as data sources for studying perinatal drug safety was investigated by determining the possibility of mother–infant linkages in these databases. Specifically, we assessed the methods used to identify the gestational period and the extent to which CDE variables, developed as part of the IMI ConcePTION project, could be incorporated.

### 2.2 Medical Databases

We selected the following Japanese medical databases: (1) administrative health care claims, (2) adverse event spontaneous reporting, and (3) birth cohort data. The Japanese Diagnosis Procedure Combination database [12], a widely used medical database in Japan, was excluded from this study because it contains only inpatient data and fully anonymized information, lacking linkage identifiers necessary to connect family members. There were other major Japanese databases that could have been potentially used to investigate infant outcomes after maternal drug exposure but they were generally difficult for external researchers to access individual‐level data and so they were excluded from this study (e.g., the Japan Environment and Children's Study [13] and The Japan Drug Information Institute in Pregnancy database [14]).

#### 2.2.1 Administrative Health Care Claims Databases

The administrative healthcare claims databases examined included: the DeSC database (DeSC Healthcare Inc.), which consists of health care claim records from the Health Insurance Association and from the National Health Insurance [15], EBM Provider [15, 16], and MDV Analyzer [17] (Medical Data Vision Co. Ltd.), JMDC claims database (JMDC Inc.), which includes health care claim records from the Health Insurance Association [15, 16], and the National Database of Health Insurance Claims (NDB), compiled by the Ministry of Health, Labour, and Welfare [15].

#### 2.2.2 Adverse Event Spontaneous Reporting Database

The database examined was the Japanese Adverse Drug Event Report (JADER) database [18, 19].

#### 2.2.3 Birth Cohort Database

The database examined was the Tohoku Medical Megabank Project Birth and Three‐Generation Cohort Study (TMM BirThree Cohort Study) [16, 20].

### 2.3 Availability of Mother–Infant Linkage

We evaluated the availability of mother‐infant linkage in each database by determining whether it included insurance registry data and family‐level unique identifiers (e.g., family identification codes shared by both mother and infant) to link maternal and infant records. Based on this assessment, databases were classified as “Available,” “Unavailable,” “Limited,” or “Not applicable.” Databases classified as “Available” contained unique identifiers that enabled deterministic mother‐infant linkage across all relevant data. Those lacking such unique identifiers were classified as “Unavailable” and excluded from further analysis. Databases were classified as “Limited” if unique identifiers for deterministic mother–infant linkage were present only for a subset of the data. “Not applicable” referred to databases where deterministic mother‐infant linkage was not possible due to the configuration of the database.

### 2.4 Identification of Pregnancies

To describe how pregnancies were identified in each mother‐infant linkable database, we reviewed previous reports on perinatal pharmacoepidemiology that used the respective database.

### 2.5 Assessment of CDE Presence Across Databases

To assess the applicability of mother‐infant linkable databases in perinatal pharmacoepidemiology, we assessed the extent to which each database captured the elements listed in the CDE variables specified for this field. The list of CDE variables comprised 98 individual data elements arranged in 14 items of related fields, as follows: (1) Database administrative details; (2) Maternal/paternal details; (3) Pregnancy details; (4) Maternal medical history; (5) Family medical and obstetric history; (6) Pregnancy medication exposure; (7) Maternal illness and obstetric complication history; (8) Details of pregnancy outcome; (9) Delivery details; (10) Live/stillborn birth outcome; (11) Live birth neonatal/infant outcome details; (12) Malformation details; (13) Long‐term child health outcome details; and (14) Long‐term neurodevelopmental outcomes. Moreover, 65 elements were identified as essential for studying pregnancy and infant outcomes, while 74 elements were considered essential for investigating long‐term childhood outcomes.

We conducted a two‐step evaluation of each database's content to determine the presence of 98 individual data elements. First, researchers experienced with each database interviewed the data managers and conducted an initial evaluation, as detailed below. Second, the research team collectively reviewed the findings and made a preliminary assessment of each database. The presence of each data element was scored based on the CDE variables, as “Yes,” “Limited,” “No,” or “NA.” “Yes” was defined as the data element being fully present; “Limited” was defined as the data element being partially present; and “No” was defined as the data element being absent. If a CDE variable was not relevant to a particular database, it was rated “NA” (e.g., primary reporter contact details in an administrative claims database). Lastly, we calculated the number and proportion of CDE variables categorized as “Yes,” “Limited,” or “NA” in each database. Coverage was defined as the combined proportion of CDE variables rated “Yes” or “Limited” divided by the total number of CDE variables assessed in that database. These calculations were performed separately for the 65 variables essential to studies on pregnancy and infant outcomes and the 74 variables essential to studies on long‐term childhood outcomes.

## 3 Results

### 3.1 Availability of Mother–Infant Linkage

Databases are described in Table 1. Based on the above‐described analysis, we determined that there were only three databases that can be considered “mother–infant linkable”: the DeSC database, the JMDC claims database, and the TMM BirThree Cohort database. Unique identifiers linking mothers to infants were classified as “Available” in the JMDC claims database and in the TMM BirThree Cohort database. Such identifiers were classified as “Limited” in the DeSC database, as some health records from the National Health Insurance did not include family‐level identifiers. The NDB, EBM Provider, and MDV Analyzer were classified as “Unavailable” due to the absence of family relationship information necessary for linking mothers and infants. The JADER database was classified as “Not applicable” because, although it contains data on adverse drug reactions, it does not separate records for mothers and infants. While JADER can support hypothesis‐generating studies by identifying pregnancy‐related reports through algorithms‐based approaches, it was excluded from the set of databases considered applicable for perinatal pharmacoepidemiologic research based on CDE variables.

**TABLE 1:** Description of medical databases and availability of mother–infant linkages.

Short name	DeSC	JMDC	NDB	EBM provider	MDV analyzer	JADER	TMM BirThree cohort
Name	DeSC database	JMDC claims database	National Database of Health Insurance Claims	EBM provider	MDV analyzer	Japanese Adverse Drug Event Report database	Tohoku Medical Megabank Project Birth and Three‐Generation Cohort database
Type of database	Administrative healthcare database	Administrative healthcare database	Administrative healthcare database	Hospital administrative database	Data service for analyzing hospital administrative database	Adverse drug event spontaneous reporting database	Cohort database
Database organizer	DeSC Healthcare Inc.	JMDC Inc.	Ministry of Health, Labour and Welfare	Medical Data Vision Co. Ltd.	Medical Data Vision Co. Ltd.	Pharmaceuticals and Medical Devices Agency	Tohoku University Tohoku Medical Megabank Organization
Number of unique identifiers	15.4 million^a^	20 million^a^	120 million^a^	48.3 million^a^	48.3 million^a^	—	74 thousand^c^
General description of the database	A database of anonymized processed information based on receipt data, annual medical checkup data, and enrollment information collected from multiple Employee Health Insurance, National Health Insurance, and Latter‐Stage Elderly Healthcare System. PRO data and life log data can be provided as options^a^	Japan's largest epidemiological database consisting of receipts, health checkup results, and subscriber registers from insurers nationwide. The database has excellent patient traceability (up to 17 years), and its population includes healthy people, making it possible to examine the prevalence and incidence of diseases. It is also linked to the PRO Panel, which enables direct approaches to patients through questionnaires and article distribution^a^	Database of health insurance claims and specific health checkups for preparation, implementation, and evaluation of medical cost optimization plan^a^	Administrative database for inpatient and outpatient consists of 519 acute (mainly considered as “advanced treatment hospitals”) hospitals in Japan^a^	A tool to analyze the MDV database. This database includes information on inpatients and outpatients from numerous Japanese acute diagnostic procedure combination hospitals^b^	An adverse drug event spontaneous reporting database published by the Pharmaceuticals and Medical Devices Agency that contains completely anonymized data reported from healthcare professionals and pharmaceutical companies	TMM BirThree Cohort Study collected in utero and subsequent exposure and outcome information, including disaster‐related information, of newborns and other family members. To resolve ‘missing heritability’ issues, the TMM BirThree Cohort Study involves plans to use this information and rich family relationship information^c^
Availability of mother–infant linkage	Limited	Available	Unavailable	Unavailable	Unavailable	Not applicable	Available
Identification of pregnancies	Using an algorithm	Using an algorithm	—	—	—	—	Directly collect

^a^ Ref. [15].

^b^ Ref. [17].

^c^ Ref. [20].

### 3.2 Identification of Pregnancies

The gestational period was estimated using an algorithm consisting of records with gestational age and records for delivery in the DeSC database [21] and JMDC claims databases [8]. In the TMM BirThree Cohort database this information was directly collected [20].

### 3.3 Assessment of CDE Presence Across Databases

The number and proportion of CDE variables categorized as “Yes,” “Limited,” or “NA” in mother–infant linkable databases are shown in Table 2. Detailed information on the presence of each individual CDE variable is provided in Table S1. The proportions of the CDE variables in each presence category were as follows: In the DeSC database, 32.7% (32/98) of the variables were present, 40.8% (40/98) were partially present, and 3.1% (3/98) were irrelevant. In the JMDC claims database, 33.7% (33/98) of CDEs were present, 39.8% (39/98) were partially present, and 3.1% (3/98) were irrelevant. In the TMM BirThree Cohort database, 84.7% (83/98) of the CDE variables were present and 8.2% (8/98) were partially present. All three mother‐infant linkable databases included all CDE variables in Items 1, 4, 7, 9, 11, 12, and 14, with each variable rated as either “Yes” (fully present) or “Limited” (partially present). For the CDE variables in Items 5, 6, 8, and 13, when the “NA” CDE variables were excluded, more than 70% of the remaining CDEs were generally found to be present across the medical databases. Finally, with respect to the CDE variables in Items 2, 3, and 10, nearly all variables were present in the TMM BirThree Cohort database, but a lower proportion was found in the DeSC and JMDC claims databases. The overall coverage of the CDE variables in the DeSC database, JMDC claims database, and TMM BirThree Cohort databases was 73.5%, 73.5%, and 92.9%, respectively.

**TABLE 2:** Presence of core data elements across medical databases.

Item	Number of CDE	DeSC database	JMDC claims database	TMM BirThree cohort
Item 1: Database administrative details	8	Yes: 25.0% (2/8) Limited: 37.5% (3/8) NA: 37.5% (3/8)	Yes: 37.5% (3/8) Limited: 25.0% (2/8) NA: 37.5% (3/8)	Yes: 100% (8/8)
Item 2: Maternal/paternal details	16	Yes: 12.5% (2/16) Limited: 18.8% (3/16)	Yes: 12.5% (2/16) Limited: 18.8% (3/16)	Yes: 87.5% (14/16)
Item 3: Pregnancy details	6	Yes: 0% (0/6) Limited: 66.7% (4/6)	Yes: 0% (0/6) Limited: 66.7% (4/6)	Yes: 83.3% (5/6) Limited:16.7% (1/6)
Item 4: Maternal medical history details	1	Yes: 100% (1/1)	Yes: 100% (1/1)	Yes: 100% (1/1)
Item 5: Family medical history and obstetric history details	10	Limited: 100% (10/10)	Limited: 100% (10/10)	Yes: 80.0% (8/10) Limited: 10.0% (1/10)
Item 6: Pregnancy medication exposure details	11	Yes: 45.5% (5/11) Limited: 36.4% (4/11)	Yes: 45.5% (5/11) Limited: 36.4% (4/11)	Yes: 45.5% (5/11) Limited: 27.3% (3/11)
Item 7: Maternal illness and obstetric complication details	3	Yes: 66.7% (2/3) Limited: 33.3% (1/3)	Yes: 66.7% (2/3) Limited: 33.3% (1/3)	Yes: 100% (3/3)
Item 8: Pregnancy outcome details	10	Yes: 40.0% (4/10) Limited: 50.0% (5/10)	Yes: 40.0% (4/10) Limited: 50.0% (5/10)	Yes: 100% (10/10)
Item 9: Delivery details	3	Yes: 33.3% (1/3) Limited: 66.7% (2/3)	Yes: 33.3% (1/3) Limited: 66.7% (2/3)	Yes: 100% (3/3)
Item 10: Live/stillborn birth outcome details	8	Yes: 12.5% (1/8) Limited: 12.5% (1/8)	Yes: 12.5% (1/8) Limited: 12.5% (1/8)	Yes: 100% (8/8)
Item 11: Live born neonatal/infant outcome details	3	Yes: 66.7% (2/3) Limited: 33.3% (1/3)	Yes: 66.7% (2/3) Limited: 33.3% (1/3)	Yes: 66.7% (2/3) Limited: 33.3% (1/3)
Item 12: Malformation details	3	Yes: 100% (3/3)	Yes: 100% (3/3)	Yes: 100% (3/3)
Item 13: Longer‐term child health outcome details	11	Yes: 81.8 (9/11) Limited: 9.1% (1/11)	Yes: 81.8 (9/11) Limited: 9.1% (1/11)	Yes: 72.7 (8/11) Limited: 18.2% (2/11)
Item 14: Longer‐term child neurodevelopmental outcome details	5	Limited: 100% (5/5)	Limited: 100% (5/5)	Yes: 100% (5/5)
Essential to collect when studying pregnancy and infant outcomes	65	Yes: 43.1% (28/65) Limited: 35.4% (23/65) NA: 4.6% (3/65)	Yes: 43.1% (28/65) Limited: 35.4% (23/65) NA: 4.6% (3/65)	Yes: 83.1% (54/65) Limited: 10.8% (7/65)
Essential to collect when studying longer term childhood outcomes	74	Yes: 40.5% (30/74) Limited: 33.8% (25/74) NA: 4.1% (3/74)	Yes: 40.5% (30/74) Limited: 33.8% (25/74) NA: 4.1% (3/74)	Yes: 83.8% (62/74) Limited: 9.5% (7/74)
Total	98	Yes: 32.7% (32/98) Limited: 40.8% (40/98) NA: 3.1% (3/98)	Yes: 33.7% (33/98) Limited: 39.8% (39/98) NA: 3.1% (3/98)	Yes: 84.7% (83/98) Limited: 8.2% (8/98)

*Note:* The items in this column correspond to table numbers cited from Richardson et al. [6].

Abbreviations: CDE, core data element; TMM BirThree Cohort, Tohoku Medical Megabank Project Birth and Three‐Generation Cohort.

In the DeSC database, of the 65 CDE variables identified as essential for studying pregnancy and infant outcomes, 43.1% (28/65) were fully present, 35.4% (23/65) were partially present, and 4.6% (3/65) were not available. Similarly, in the JMDC claims database, 43.1% (28/65) of the CDE variables were fully present, 35.4% (23/65) were partially present, and 4.6% (3/65) were deemed not relevant. In the TMM BirThree Cohort database, 83.1% (54/65) of the CDE variables were present and 10.8% (7/65) were partially present.

For the 74 CDE variables identified as essential for studying long‐term childhood outcomes, the DeSC database contained 40.5% (30/74) of the variables, 33.8% (25/74) were partially present, and 4.1% (3/74) were not relevant. The JMDC claims database showed the same proportions. In the TMM BirThree Cohort database, 83.8% (62/74) were present and 9.5% (7/74) were partially present.

## 4 Discussion

We focused on databases suitable for studying infant outcomes as these require mother–infant linkages. In this study, we evaluated the suitability (fitness for purpose) of Japanese medical databases for conducting perinatal pharmacoepidemiologic research on infant outcomes following maternal drug exposure. Our findings revealed that their suitability varied across databases, owing to differences in data type and quality. There were only three linkable mother‐infant databases. Of these, the DeSC and JMDC claims databases are administrative health care claims databases, in which the gestational period was estimated using records related to delivery. The coverage of CDE was 73.5% in the DeSC database (Yes: 32.7%, Limited: 40.8%) and 73.5% in the JMDC claims database (Yes: 33.7%, Limited: 39.8%). In the TMM BirThree Cohort database, where gestational age was directly obtained from participants, CDE coverage was 92.9% (Yes: 84.7%, Limited: 8.2%).

The three databases considered applicable to perinatal pharmacoepidemiology varied in their characteristics. The JMDC claims database enables mother‐infant linkage via unique ID, and several studies using this feature for perinatal pharmacoepidemiology have been reported [8, 9, 10, 11]. In contrast, the DeSC database is limited to data obtained from the National Health Insurance system. Mother‐infant linkage was limited in this database because information on family relationships is voluntarily submitted by the insured under the National Health Insurance program. Although the success rate of linkage could not be calculated, data from April 2014 to August 2021 included 27 370 pregnancy episodes for which mother–child linkage was achieved [21]. The NDB, EBM provider, and MDV analyzer, despite being large‐scale medical databases with a higher number of unique identifiers than other sources, do not support mother–infant linkage. Establishing such linkage in these databases would enable large‐scale perinatal pharmacoepidemiologic research in Japan. As in other areas of pharmacoepidemiology, the JADER database may be useful in perinatal research for hypothesis generation through signal detection [22, 23, 24].

Accurate identification of gestational age is crucial in perinatal pharmacoepidemiology, as fetus drug effects can vary depending on the timing of exposure [25]. In the present study, we reviewed the literature to understand how pregnancies are identified in various databases. Administrative healthcare claims databases do not include conception dates, as this information is unrelated to insurance claims. Therefore, gestational age must be estimated based on billing data related to delivery. In the JMDC claims database, pregnancy onset and delivery dates were estimated using an algorithm [26], with a reported positive predictive value exceeding 90% within ±7 days of the actual conception date. The DeSC database also estimated gestational periods, but details were limited to a conference presentation, and no further information was available [21]. In contrast, the TMM BirThree cohort study collected gestational age data directly from mothers, making its estimates more reliable for evaluating the timing of drug exposure during pregnancy compared to claims‐based databases. Despite differences in how gestational age is determined, no formal validation studies or reliability assessments of pregnancy identification methods have been conducted for these databases. For databases lacking any published pregnancy‐related studies, the methods for identifying pregnancies remain unknown. Further research is needed to validate pregnancy identification approaches in Japanese medical databases.

The presence of CDE variables also varied across databases. The TMM BirThree cohort study database was the most comprehensive, covering nearly all CDE variables and thus representing a suitable resource for perinatal pharmacoepidemiology. In contrast, approximately 40% of the CDE variables were categorized as “Limited” in both the DeSC and JMDC claims databases. This is not unexpected, as both rely on secondary data from insurance receipts, which are generally less detailed and of lower quality than the primary data used in research cohorts. Variables that are not typically included in insurance claims, such as those categorized in item 2 or 10, were less likely to be available in the DeSC and JMDC databases. Nevertheless, these claims databases offer the advantage of large sample sizes (Table 1). Although the TMM BirThree cohort database has limited enrollment, making it more difficult to obtain a sufficient sample size for certain analyses, the scale of the DeSC and JMDC databases allows for broader epidemiological studies. In addition, while the TMM BirThree Cohort is arguably the most valid resource among Japanese databases for assessing fetal effects of medication during pregnancy, its accessibility is more limited. Access requires a formal application and review process, which can result in significant delays [27]. In contrast, other databases are commercially operated and more readily accessible to independent researchers. These differences illustrate the trade‐off between data validity and feasibility when selecting a database for perinatal pharmacoepidemiology research.

This study has two main limitations. First, the initial evaluation of CDE variable presence in mother‐infant linkable databases may have been influenced by the subjective judgment of the researchers. However, we conducted a second‐stage evaluation involving all authors to eliminate bias. Second, the CDE variables used, developed under the IMI ConcePTION project, represent just one framework for assessing database suitability. Other studies have evaluated databases from different perspectives [16]. Nevertheless, CDE variables provide a useful framework for detailed and standardized data collection and referencing them remains a primary step in evaluating the suitability of medical databases for perinatal pharmacoepidemiology. A key strength of this study is its comprehensive evaluation of diverse database types, such as administrative claims data, adverse drug event reporting systems, and birth cohort studies. Further investigations should include all available Japanese medical databases to provide a more complete understanding of their utility in perinatal pharmacoepidemiology.

## 5 Conclusion

Our analysis shows that some Japanese medical databases contain a high proportion of essential data for evaluating perinatal drug safety. However, careful selection is necessary, as the availability of relevant data varies across databases.

### 5.1 Plain Language Summary

Taking medicine during pregnancy requires careful consideration, as certain drugs may pose risks to the fetus, including birth defects. To protect maternal and fetal health, it is essential to monitor medication use and investigate its effects during pregnancy. However, in Japan, the collection of such data has been limited due to underdeveloped research infrastructure. This study evaluated seven medical databases in Japan to assess their suitability for investigating the safety of medication use during pregnancy. Among them, three databases (the DeSC database, JMDC claims database, and the Tohoku Medical Megabank Project Birth and Three‐Generation Cohort Study) were capable of linking maternal and infant records. These three databases contained a high proportion of the core data elements required for perinatal pharmacoepidemiologic research: the DeSC and JMDC claims databases included approximately 74% of these key variables, while the Tohoku Medical Megabank Project Birth and Three‐Generation Cohort Study database included about 93%. These findings suggest that certain Japanese medical databases hold significant potential for studying the safety of medication use during pregnancy.

## Author Contributions

T.O. was responsible for organizing and coordinating the study. S.H. was the chief investigator responsible for the data analysis. S.H., T.S., Y.O., and T.O. designed the study. M.T., Y.O., and T.O. interviewed the data holders of each medical database and conducted the first evaluation of data presence for the CDE variables. S.H., T.S., I.S., Y.O., and T.O. wrote the manuscript. All authors contributed to the secondary evaluation of data presence for CDE variables, writing of the final manuscript, and management or administration of this study.

## Ethics Statement

In each of the databases analyzed, although patient records were assigned identification numbers, the actual identity of the patient (name, address, etc.) was not visible to the researchers. Therefore, patient consent was not needed in this study.

## Conflicts of Interest

The authors declare no conflicts of interest.

## Supporting information


**Table S1:** Details of the core data elements for perinatal pharmacoepidemiology in each database.

## Data Availability

The authors have nothing to report.
