# Supplementary material for: Suitability of Japanese Medical Databases for Studies on Infant Outcomes After Maternal Drug Exposure: An Evaluation Based on Core Data Elements
Source: Pharmacoepidemiol Drug Saf. 2025 Nov 12;34(11):e70264. doi: 10.1002/pds.70264 (PMC12611501; doi:10.1002/pds.70264)
Supplement: Supplementary file 1 — Table S1: Details of the core data elements for perinatal pharmacoepidemiology in each database. [file PDS-34-e70264-s001.docx]

Table S1 Details of the core data elements for perinatal pharmacoepidemiology in each database.

| Item | No. | CDE Item | Essential to collect when studying pregnancy and infant outcomes | Essential to collect when studying longer term childhood outcomes | DeSC database | JMDC claims database | TMM BirThree Cohort |
| --- | --- | --- | --- | --- | --- | --- | --- |
| Item 1: Database administrative details | 1 | Pregnancy exposure | Yes | Yes | Limited | Limited | Yes |
|  | 2 | Mother case identifier | NA | NA | Limited | Limited | Yes |
|  | 3 | Baby case identifier | NA | NA | Yes | Yes | Yes |
|  | 4 | Mother Baby case link identifier | NA | NA | Limited | Yes | Yes |
|  | 5 | Primary reporter type | Yes | Yes | NA | NA | Yes |
|  | 6 | Primary reporter contact details | Yes | Yes | NA | NA | Yes |
|  | 7 | Initial report date | Yes | Yes | NA | NA | Yes |
|  | 8 | Prospective status | Yes | Yes | Yes | Yes | Yes |
| Item 2: Maternal/paternal details | 1 | Maternal date of birth | Yes | Yes | Yes | Yes | Yes |
|  | 2 | Maternal age at last menstrual period (LMP) | Yes | Yes | Limited | Limited | Yes |
|  | 3 | Household income | No | Yes | No | No | Yes |
|  | 4 | Maternal education | No | Yes | No | No | Yes |
|  | 5 | Paternal education | No | No | No | No | Yes |
|  | 6 | Maternal IQ | No | No | No | No | No |
|  | 7 | Maternal ethnicity | No | No | No | No | Yes |
|  | 8 | Consanguinity | No | Yes | Limited | Limited | Yes |
|  | 9 | Maternal Height | No | Yes | No | No | Yes |
|  | 10 | Maternal Weight prepregnancy | No | No | No | No | Yes |
|  | 11 | Maternal BMI pre-pregnancy | Yes | Yes | No | No | Yes |
|  | 12 | Maternal country of residence | No | Yes | Yes | Yes | Yes |
|  | 13 | Smoking in pregnancy | No | Yes | No | No | Yes |
|  | 14 | Alcohol in pregnancy | No | Yes | No | No | Yes |
|  | 15 | Illicit drugs in pregnancy | No | Yes | No | No | No |
|  | 16 | Folic acid use | No | Yes | Limited | Limited | Yes |
| Item 3:  Pregnancy details | 1 | Date of LMP | Yes | Yes | Limited | Limited | Limited |
|  | 2 | Expected date of delivery (EDD) | Yes | Yes | No | No | Yes |
|  | 3 | Source of directly reported EDD | Yes | Yes | No | No | Yes |
|  | 4 | Assisted conception | No | No | Limited | Limited | Yes |
|  | 5 | Plurality | Yes | Yes | Limited | Limited | Yes |
|  | 6 | Prenatal test(s) | Yes | Yes | Limited | Limited | Yes |
| Item 4:  Maternal medical history details | 1 | Maternal prepregnancy medical conditions (history) | Yes | Yes | Yes | Yes | Yes |
| Item 5:  Family medical history and obstetric history details | 1 | Family history of congenital anomalies | No | No | Limited | Limited | Yes |
|  | 2 | Relevant family history of genetic conditions | No | No | Limited | Limited | Yes |
|  | 3 | Relevant family history of learning disability or of neurodevelopmental disorders | No | No | Limited | Limited | Yes |
|  | 4 | Relevant family history of diseases with onset in childhood | No | No | Limited | Limited | Limited |
|  | 5 | Number of previous pregnancies | No | No | Limited | Limited | Yes |
|  | 6 | Number of previous live births | No | No | Limited | Limited | Yes |
|  | 7 | Number of previous spontaneous abortions | No | No | Limited | Limited | Yes |
|  | 8 | Number of previous induced terminations | No | No | Limited | Limited | Yes |
|  | 9 | Number of previous stillbirths | No | No | Limited | Limited | Yes |
|  | 10 | Number of previous pregnancies with congenital anomalies | No | No | Limited | Limited | No |
| Item 6: Pregnancy medication exposure details | 1 | Drug name(s) | Yes | Yes | Yes | Yes | Yes |
|  | 2 | Drug start date | Yes | Yes | Yes | Yes | No |
|  | 3 | Drug stop date | Yes | Yes | No | No | No |
|  | 4 | Drug indication(s) | Yes | Yes | No | No | No |
|  | 5 | Peri-LMP exposure | Yes | Yes | Limited | Limited | Limited |
|  | 6 | Trimester 1 exposure | Yes | Yes | Limited | Limited | Yes |
|  | 7 | Trimester 2 exposure | Yes | Yes | Limited | Limited | Yes |
|  | 8 | Trimester 3 exposure | Yes | Yes | Limited | Limited | Limited |
|  | 9 | Route of exposure | Yes | Yes | Yes | Yes | Limited |
|  | 10 | Dose per use | Yes | Yes | Yes | Yes | Yes |
|  | 11 | Frequency of use | Yes | Yes | Yes | Yes | Yes |
| Item 7:  Maternal illness and obstetric complication details | 1 | Maternal medical conditions arising in pregnancy | Yes | Yes | Yes | Yes | Yes |
|  | 2 | Maternal postpartum complications | No | Yes | Yes | Yes | Yes |
|  | 3 | Maternal death | Yes | Yes | Limited | Limited | Yes |
| Item 8:  Pregnancy outcome details | 1 | Pregnancy outcome collection status | Yes | Yes | Limited | Limited | Yes |
|  | 2 | Date of end of pregnancy | Yes | Yes | Limited | Limited | Yes |
|  | 3 | Gestational age at end of pregnancy | Yes | Yes | No | No | Yes |
|  | 4 | Induced termination | Yes | Yes | Limited | Limited | Yes |
|  | 5 | Ectopic pregnancy | Yes | Yes | Yes | Yes | Yes |
|  | 6 | Stillbirth | Yes | Yes | Limited | Limited | Yes |
|  | 7 | Spontaneous abortion | Yes | Yes | Limited | Limited | Yes |
|  | 8 | Molar pregnancy | Yes | Yes | Yes | Yes | Yes |
|  | 9 | Blighted ovum | Yes | Yes | Yes | Yes | Yes |
|  | 10 | Live birth | Yes | Yes | Yes | Yes | Yes |
| Item 9:  Delivery details | 1 | Labour onset | No | No | Limited | Limited | Yes |
|  | 2 | Mode of delivery | No | No | Limited | Limited | Yes |
|  | 3 | Maternal delivery complications | No | No | Yes | Yes | Yes |
| Item 10:  Live/stillborn birth outcome details | 1 | Gestational timing of live/stillborn offspring | Yes | Yes | Limited | Limited | Yes |
|  | 2 | Infant birth weight | Yes | Yes | No | No | Yes |
|  | 3 | Infant sex | Yes | Yes | Yes | Yes | Yes |
|  | 4 | Infant head circumference | Yes | No | No | No | Yes |
|  | 5 | Infant birth length | No | No | No | No | Yes |
|  | 6 | Small for Gestational Age at delivery | Yes | Yes | No | No | Yes |
|  | 7 | Large for Gestational Age at Delivery | Yes | Yes | No | No | Yes |
|  | 8 | Apgar score | No | No | No | No | Yes |
| Item 11:  Live born neonatal/infant outcome details | 1 | Complications in the first year of life | Yes | Yes | Yes | Yes | Yes |
|  | 2 | Postnatal death of live born infant | Yes | Yes | Yes | Yes | Yes |
|  | 3 | Product/diseasespecific outcomes | Yes | Yes | Limited | Limited | Limited |
| Item 12:  Malformation details | 1 | Congenital anomaly | Yes | Yes | Yes | Yes | Yes |
|  | 2 | Details of all congenital anomaly(ies) | Yes | Yes | Yes | Yes | Yes |
|  | 3 | Infant malformation case classification | Yes | Yes | Yes | Yes | Yes |
| Item 13:  Longer-term child health outcome details | 1 | Infant/child medical problems | Yes | Yes | Yes | Yes | Yes |
|  | 2 | Infant/child specialist visit | Yes | Yes | Limited | Limited | Limited |
|  | 3 | Infant/child medication | Yes | Yes | Yes | Yes | Limited |
|  | 4 | Infant/child professional concerns | Yes | Yes | No | No | No |
|  | 5 | Infant/child vision difficulties | Yes | Yes | Yes | Yes | Yes |
|  | 6 | infant/child hearing difficulties | Yes | Yes | Yes | Yes | Yes |
|  | 7 | Infant/child toileting difficulties | Yes | Yes | Yes | Yes | Yes |
|  | 8 | Infant/child eczema/ skin conditions | Yes | Yes | Yes | Yes | Yes |
|  | 9 | Infant/child asthma/ respiratory conditions | Yes | Yes | Yes | Yes | Yes |
|  | 10 | Infant/child congenital heart disease | Yes | Yes | Yes | Yes | Yes |
|  | 11 | Infant/child food allergies | Yes | Yes | Yes | Yes | Yes |
| Item 14:  Longer-term child neurodevelopmental outcome details | 1 | Child cognitive functioning | Yes | Yes | Limited | Limited | Yes |
|  | 2 | Child motor functioning | Yes | Yes | Limited | Limited | Yes |
|  | 3 | Child language functioning | Yes | Yes | Limited | Limited | Yes |
|  | 4 | Child social functioning | Yes | Yes | Limited | Limited | Yes |
|  | 5 | Child behaviour and emotional functioning | Yes | Yes | Limited | Limited | Yes |
| The items in this column correspond to table numbers cited from Richardson JL, et al. Drug Saf. 2023;46:479-491.  CDE: Core data element  TMM BirThree Cohort: Tohoku Medical Megabank Project Birth and Three-Generation Cohort | | | | | | | |
